# FGF‐stimulated tendon cells embrace a chondrogenic fate with BMP7 in newt tissue culture

**DOI:** 10.1111/dgd.12913

**Published:** 2024-02-11

**Authors:** Nao Sugiura, Kiyokazu Agata

**Affiliations:** ^1^ Department of Basic Biology The Graduate University for Advanced Studies (SOKENDAI) Okazaki Japan; ^2^ Laboratory for Regenerative Biology National Institute for Basic Biology (NIBB) Okazaki Japan

**Keywords:** FGF, joint regeneration, newt, tendons, tissue culture

## Abstract

Newts can regenerate functional elbow joints after amputation at the joint level. Previous studies have suggested the potential contribution of cells from residual tendon tissues to joint cartilage regeneration. A serum‐free tissue culture system for tendons was established to explore cell dynamics during joint regeneration. Culturing isolated tendons in this system, stimulated by regeneration‐related factors, such as fibroblast growth factor (FGF) and platelet‐derived growth factor, led to robust cell migration and proliferation. Moreover, cells proliferating in an FGF‐rich environment differentiated into Sox9‐positive chondrocytes upon BMP7 introduction. These findings suggest that FGF‐stimulated cells from tendons may aid in joint cartilage regeneration during functional elbow joint regeneration in newts.

## Graphical abstract

A serum‐free tissue culture system for tendons with regeneration‐related factors was established to explore cell dynamics during joint regeneration in newts. Our findings suggest that FGF‐stimulated cells from tendons may aid in joint cartilage regeneration during functional elbow joint regeneration in newts.

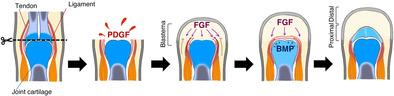

## 1 INTRODUCTION

A joint is the junction of bone with a complementary morphology between opposing bony tissues, with their surfaces covered with cartilage (joint cartilage). A joint is covered by a pouch‐like articular capsule, the inner synovial membrane that secretes joint fluid and facilitates smooth movement. Ligaments connect the bony tissues to each other and muscles are connected to the bony tissues via tendons, allowing the joints to flex and extend functionally (Benjamin et al., 2008; Rumian et al., 2007). According to various studies, newts and other salamanders have a very high regenerative ability (Agata & Inoue, 2012; Inoue et al., 2012; Iten & Bryant, 1973; Joven et al., 2019; Kaucka et al., 2022; Muneoka & Bryant, 1982; Simon & Tanaka, 2012; Urata et al., 2018), and they can regenerate functional limb structures, including joints, irrespective of which part of the limb is amputated. Regeneration occurs when the epithelium covering the wound surface thickens and forms a structure called a blastema (Endo et al., 2004), which eventually elongates during regeneration. In the case of joint regeneration in newts, primary ossification of chondrocytes regenerates hard bone and joint cartilage, whereas secondary ossification does not occur. Thus, the newt humerus retains a much larger area of joint cartilage than mammals, implying that the lack of secondary ossification and the large area of joint cartilage may be important in joint regeneration (Tsutsumi et al., 2015). However, it has been reported that when the joint cartilage of the humerus is removed by amputation at the level of the elbow joint, the complementary proximal joint cartilage (concave surface) cannot regenerate, but the structure of the distal portion can be fully regenerated (Tsutsumi et al., 2015). This suggests that the joint cartilage and surrounding tissues of the residual humerus are key elements in the regeneration of the newt elbow joint.

In contrast, even among amphibians, there are differences in regenerative ability between species. Anuran amphibians, such as frogs and toads, cannot regenerate joints after metamorphosis. However, they form a blastema, which can extend a cartilaginous rod‐like structure called a “spike” without limb patterning (Hayashi et al., 2015; Satoh et al., 2005). Our previous study demonstrated the formation of a spike, including a functional joint, in *Xenopus laevis* when amputation was performed at the upper‐arm elbow joint level (Tsutsumi et al., 2016). The cartilage in these residual and regenerated areas was joined properly, and the regenerating joint cartilage showed expression of both *scx* and *sox9* (specific marker genes for tendons and cartilage, respectively) (Tsutsumi et al., 2016). These results suggest that the residual tendons surrounding the joint cartilage of the stump might harbor cells contributing to cartilage regeneration.

Although the origin of cells contributing to regeneration and the mechanisms of joint regeneration are still not clearly understood (Kragl et al., 2009; Satoh et al., 2022; Tsutsumi et al., 2015), there are many reports on the involvement of certain factors associated with limb and joint regeneration in amphibians. One promising candidate is the group of fibroblast growth factors (FGFs), which are known as important signaling molecules for blastema formation (Aztekin et al., 2021; Maddaluno et al., 2017; Makanae et al., 2014; Okumura et al., 2019). FGFs are produced by the nerve and wound epithelium (Maddaluno et al., 2017; Makanae & Satoh, 2012). Bone morphogenic protein (BMP) signaling, along with FGF signaling, is essential for limb regeneration (Satoh et al., 2022), and has been shown to cooperate with FGF to induce regeneration in *Ambystoma mexicanum* (axolotl) limbs (Makanae et al., 2014; Makanae et al., 2016; Vieira et al., 2019). It can also induce joint and joint‐like structures in *X. laevis* limb regeneration (Satoh et al., 2005). Another candidate, platelet‐derived growth factor (PDGF), is a potent inducer of fibroblast migration that is required for blastema formation. Studies have shown that inhibition of PDGF signaling blocks the migration of soft connective tissue cells and prevents blastema formation during axolotl limb regeneration (Currie et al., 2016). Thus, FGF, BMP, and PDGF may function as regeneration inducers during joint regeneration in amphibians. Moreover, in *X. laevis*, the remaining stump tissues may be activated by signals derived from the blastema to achieve proper tissue connection for functional joint regeneration (Matsubara et al., 2023).

Furthermore, it was necessary to identify which tissue‐derived cells in the remaining area contribute to the regeneration of articular cartilage during joint regeneration. In axolotl, the regenerating skeleton originates from several cellular sources, including mainly periskeletal cells and dermal fibroblasts, and tracing experiments revealed no lineage‐specific stem cells for cartilage and bones; instead periskeletal cells extended the bone by generating an ossifying cartilaginous callus (Currie et al., 2016; Gerber et al., 2018; Kaucka et al., 2022). Therefore, the remaining periarticular tissue cells may be a source of the regenerated joint cartilage. Tendons are connective tissues that connect to the articular cartilage. Tendon cells originate from the lateral plate mesodermal lineage and differentiate from the chondrocytes of the same origin (Pryce et al., 2009). As tendon and cartilage progenitor cells co‐express *scx* and *sox9* (*scx* and *sox9* double‐positive cells) during joint regeneration in *X. laevis* (Tsutsumi et al., 2016), cartilage is likely to be regenerated by tendon cells that have de‐differentiated and transdifferentiated to chondrocytes. Therefore, to identify the origin of the cells contributing to joint regeneration, we focused on tendon tissues in the remaining area. As an experimental system to investigate the origin of cells contributing to joint regeneration and the role of regeneration‐related factors, we established a tissue culture system that allowed direct observation of migrating and differentiating cells from the tissue in newts (*Pleurodeles waltl*). Newts, in particular, have a high regenerative capacity and are easy to handle (Hayashi et al., 2013). It has attracted attention as a new model organism for organ regeneration and was therefore used in this study. Tissue cultures of tendons were used to examine the roles of regeneration‐related factors, proliferative capacity, and chondrogenic differentiation potential. Our findings provide interesting insights into the origin of cells that contribute to the regeneration of joint cartilage.

## 2 MATERIALS AND METHODS

### 2.1 Animals

Iberian ribbed newts (*P. waltl*) were purchased from Hiroshima University Amphibian Research Center (Hiroshima, Japan). The newts used in this study were less than 4 months old and 8 cm long. They were kept in tap water at 25–26°C and anesthetized with 0.2% ethyl 3‐aminobenzoate methanesulfonate (MS‐222) for surgical amputation. All animal experiments were conducted according to a protocol approved by the National Institute of Natural Sciences (23A011).

### 2.2 Tissue culture

After the limbs were amputated at the shoulder level, the tissue was sterilized in 70% ethanol for 10 s and washed twice with 70% PBS. Tendons were dissected from the upper‐arm muscles of the forelimb elbow joint after peeling off the skin using pin sets in 500 μg/mL gentamicin in 70% PBS solution. Each tissue was placed on 24‐well culture plates coated with iMatrix 511‐silk (Nippi), which is a cell culture substrate purified from silkworm cocoon in which human Laminin‐511 fragment E8 genes are incorporated. To adhere the tissue to the surface of the wells, they were embedded in collagen gel made of Type IA collagen (Nitta Gelatin Inc.), 10× αMEM (Wako‐Fuji film), and reconstitution buffer (Nitta Gelatin Inc.) and then kept stationary in an incubator for 30 min at 25°C with 5.0% CO_2_. After the collagen gel hardened, the medium was added to each well and samples were incubated under the same conditions.

### 2.3 Culture medium

The base culture medium consisted of 0.8× N2B27 culture medium, 25% D‐MEM/Ham's F‐12 (Gibco), 25% Neurobasal Medium (Gibco), 0.8× N‐2 Supplement (100×, Thermo Fisher Scientific), 0.8× B‐27 Supplement without Vitamin‐A (50×, Thermo Fisher Scientific), 1× Penicillin–Streptomycin (100×, Gibco), 0.1 mM β‐mercaptoethanol (Nacalai Tesuque), and 0.1 mM l‐alanyl‐l‐glutamine (Wako‐Fuji film). Growth factors such as FGF2 (Wako‐Fuji film) and FGF8 (Wako‐Fuji film) were added to the medium at a concentration of 100 ng/μL. PDGF‐BB (Wako‐Fuji film) was added to the medium at a concentration of 15 ng/μL. Positive control medium consisted of 25% D‐MEM/Ham's F‐12 (Gibco), 25% Neurobasal Medium (Gibco), 10% fetal bovine serum (FBS), 1× Penicillin–Streptomycin (100×, Gibco), 0.1 mM β‐mercaptoethanol (Nacalai Tesuque), and 0.1 mM l‐alanyl‐l‐glutamine (Wako‐Fuji film). The differentiation‐inducing medium consisted of 0.8× N2B27 culture medium, 25% D‐MEM/Ham's F‐12 (Gibco), 25% Neurobasal Medium (Gibco), 0.8× N‐2 Supplement (100×, Thermo Fisher Scientific), 0.8× B‐27 Supplement (50×, Thermo Fisher Scientific), 1× Penicillin–Streptomycin (100×, Gibco), 0.1 mM β‐mercaptoethanol (Nacalai Tesuque), and 0.1 mM l‐alanyl‐l‐glutamine (Wako‐Fuji film) with BMP7. BMP7 (Wako‐Fuji film) was added to the medium at a concentration of 100 ng/μL.

### 2.4 EdU assay

Following procedures were carried out on a shaker. EdU stock solution (10 mM) was added to the fresh culture medium without any growth factor at a final concentration of 10 μM, and samples were incubated in EdU‐containing medium for 72 h at 25°C with 5.0% CO_2_. The tissues were fixed with 4% paraformaldehyde in PBS for 15 min at room temperature. A Click‐iT EdU Alexa fluor 594 imaging kit (Invitrogen) was used to visualize proliferating cells. The tissue samples were washed twice with PBS and twice with 3% FBS in PBS for 5 min at room temperature. For membrane permeabilization, samples were incubated in PBS containing 0.5% Triton X‐100 for 20 min at room temperature. Samples were washed twice with 3% FBS in PBS for 5 min at room temperature. A Click‐iT reaction cocktail (1× Click‐iT reaction buffer, CuSO_4_, Alexa Fluor azide, reaction buffer additive) was added and samples were incubated for 1 h at room temperature in the dark. The cells were further washed with 3% FBS in PBS and PBS for 5 min at room temperature. Hoechst 33342 (1:2000) solution was added and samples were incubated for 30 min at room temperature in the dark. Images were acquired using a confocal laser microscope (LSM980, Zeiss). Cells were counted using ImageJ and Fiji‐3.

### 2.5 Immunocytochemistry

The following procedures were carried out on a shaker. Tissues were fixed with 4% paraformaldehyde in PBS for 15 min at room temperature. Then they were washed twice with PBS and twice with 1% BSA in PBST for 5 min at room temperature. For membrane permeabilization, the tissues were kept in 0.5% Triton X‐100 in PBS for 20 min at room temperature. Samples were again washed twice with 1% BSA in PBST for 5 min at room temperature. Subsequently, 1% BSA in PBST was added and the tissues were incubated for 1 h at room temperature. The solution was removed, the primary antibody solution was added, and samples were incubated overnight at 4°C. Rabbit anti‐Sox9 (1:1000, Cell Signaling, D8G8H/82630 S) was used as the primary antibody (Kaucka et al., 2022). The cells were washed twice with PBS and once with 1% BSA in PBST for 5 min at room temperature. The secondary antibody solution was added and samples were incubated for 1 h at room temperature in the dark. Samples were washed twice with PBS for 5 min at room temperature. Finally, Hoechst 33342 (1:2000) solution was added and samples were incubated for 30 min at room temperature in the dark. Samples were washed again twice with PBS for 5 min under dark conditions at room temperature.

### 2.6 Quantitative RT‐PCR

For quantitative reverse transcription PCR (RT‐PCR), total RNA was extracted using the RNeasy Micro Kit (Qiagen) and reverse‐transcribed using the Prime Script™ RT reagent kit with g‐DNA Eraser (Perfect Real Time, Takara). Quantitative RT‐PCR was performed using a Light Cycler® 96 system (Roche) and THUNDERBIRD® Next SYBR® qPCR Mix (Toyobo). The Ct values for each gene were standardized using *gapdh* and quantified using the 2^−ΔΔCt^ method. All primer sets were designed using primer3 software.

List of primer sets used:

p.waltl_Gapdh FW: TGACGATCACTCATCAATCTTTG

p.waltl_Gapdh RV: GTTGACTGTAACCAAATTCATTGTC

p.waltl_Col1a1 FW: AAGGAACCAGGAAGAACCCT

p.waltl_Col1a1 RV: CAGCCCTGGTTAGGATCAAT

p.waltl_Col2a1 FW: GACTTAGGTGAGAAAGGCCCAGAAG

p.waltl_Col2a1 RV: GGAGGACCAGATTCACCCTTCTC

p.waltl_Sox9 FW: CTACAACTCTACCTACCCTACTATTACACG

p.waltl_Sox9 RV: GTTCATGTAGGAGAAAGTGGAGTAGAGGTT

p.waltl_Scx FW: CTCCAGCTACATCTCGCACCTG

p.waltl_Scx RV: GTCTCTGTCTTTGCTCTGCTTGC

p.waltl_Tnmd FW: GGATTCCAATGGAGAAACCCATA

p.waltl_Tnmd RV: TCAGGTCCTTCCTCAAAATCCAT

## 3 RESULTS

### 3.1 Development of a new tissue culture system

**FIGURE 1:**
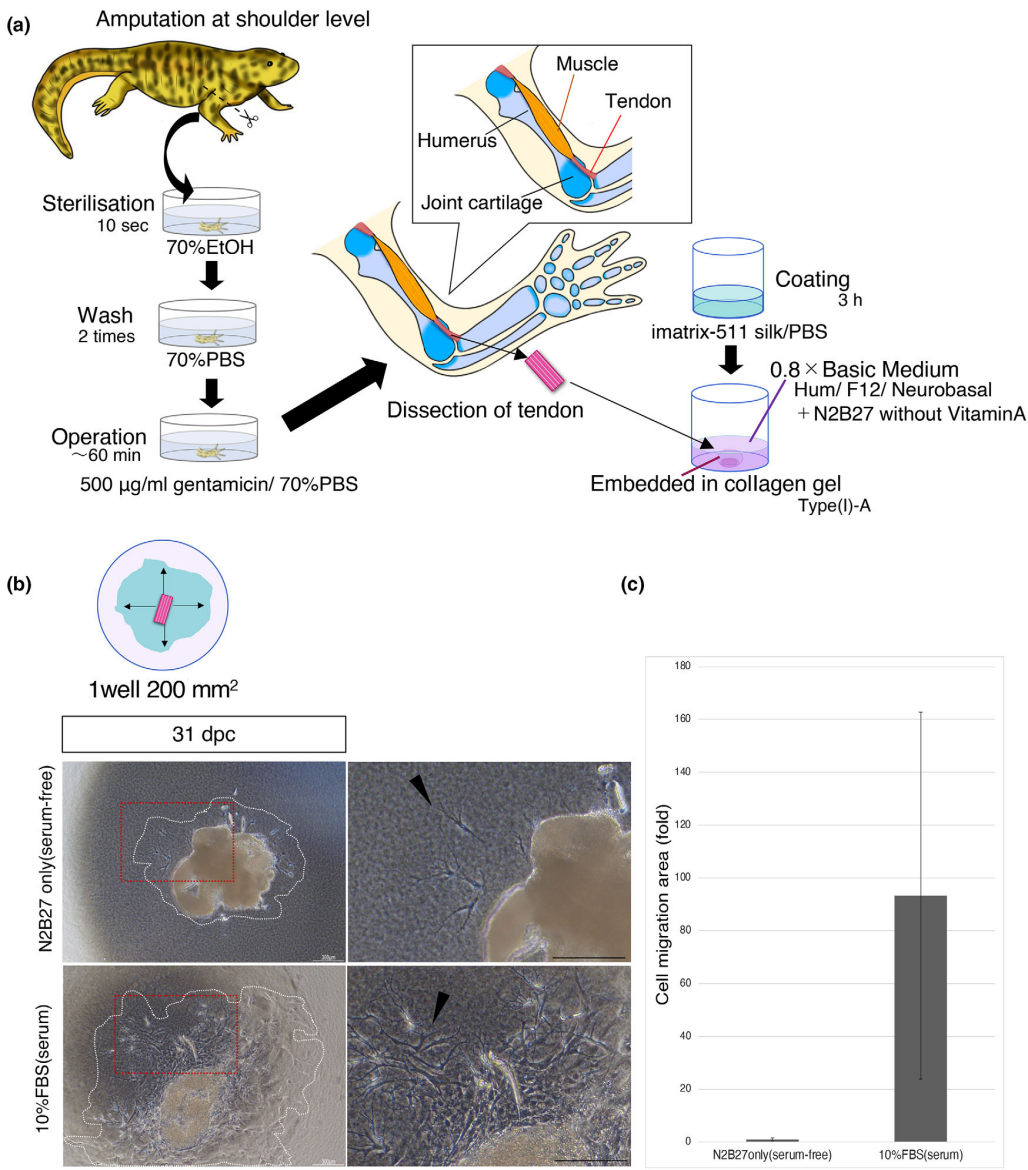
Development of a new tissue culture system. (a) Schematic of the tissue culture system. Tendons are dissected from the upper‐arm muscles in the elbow joint, placed in a collagen gel, and cultured in a medium with regeneration‐related factors. (b) Comparison of cell migration in serum‐containing and serum‐free medium in tendon tissue on day 31. The upper figure shows a schematic of the cell migration area (one well is 200 mm^2^). The cell migration area is surrounded by a white dotted line. The lower figures show the actual cell migration at 31 days post‐cultured (dpc) in the bright field image. The right‐hand figures show a magnified view of the area surrounded by the red box in the left‐hand figure. Black arrows indicate fibroblast‐like cells. Scale bar, 300 μm. (c) Graph showing the migration area using the serum‐free medium as the reference value, illustrating the impact of 10% FBS in the medium. The vertical axis represents the factor by which the migration range is increased, using serum‐free medium as the reference. On average, the cell migration range was 93 times higher than that in serum‐free medium. *n* = 3. Bars: SE.

To date, no standardized conditions have been developed for *P. waltl* tissue culture systems. First, we developed a culture system to visualize the migration of isolated tissue‐derived cells (Figure 1a). Although serum media with 10% FBS are commonly used in culture systems, we adopted a serum‐free medium using N2B27 to add regeneration‐related factors that might be involved in newt joint regeneration and clarify their role during regeneration. It is difficult to induce cell proliferation and adhesion in serum‐free media; therefore, we explored different coating reagents. Tendon cells were found to take more than a month for cell proliferation to occur and 2 months for cell migration when using gelatin coating. Newts exhibit slow cell proliferation owing to their large genome size. The best cell adhesion and migration conditions were observed with serum‐free coatings on a laminin‐based cell culture substrate (iMatrix 511‐silk). Using this coating reagent, we could shorten the time from cell inoculation to cell migration to 10–14 days (instead of 2 months). Furthermore, the tissue culture system allowed long‐term culture for over 2 months. The comparison of serum‐free and 10% FBS‐containing media showed a clear difference in cell migration, expansion range, and cell proliferation after 31 days (Figure 1b). Long fibroblast‐like cells with pseudopodia began to migrate from the tendon tissue (black arrowheads in Figure 1b). The range of cell migration was, on average, 93 times larger than that in serum‐free medium (Figure 1c). In samples without serum, there was no noticeable variation in cell emergence and migration. However, when serum was added, cells exhibited active migration and spreading; nevertheless, there was variability in the extent of this spreading among samples.

### 3.2 Effects of regeneration‐related factors on cell migration

**FIGURE 2:**
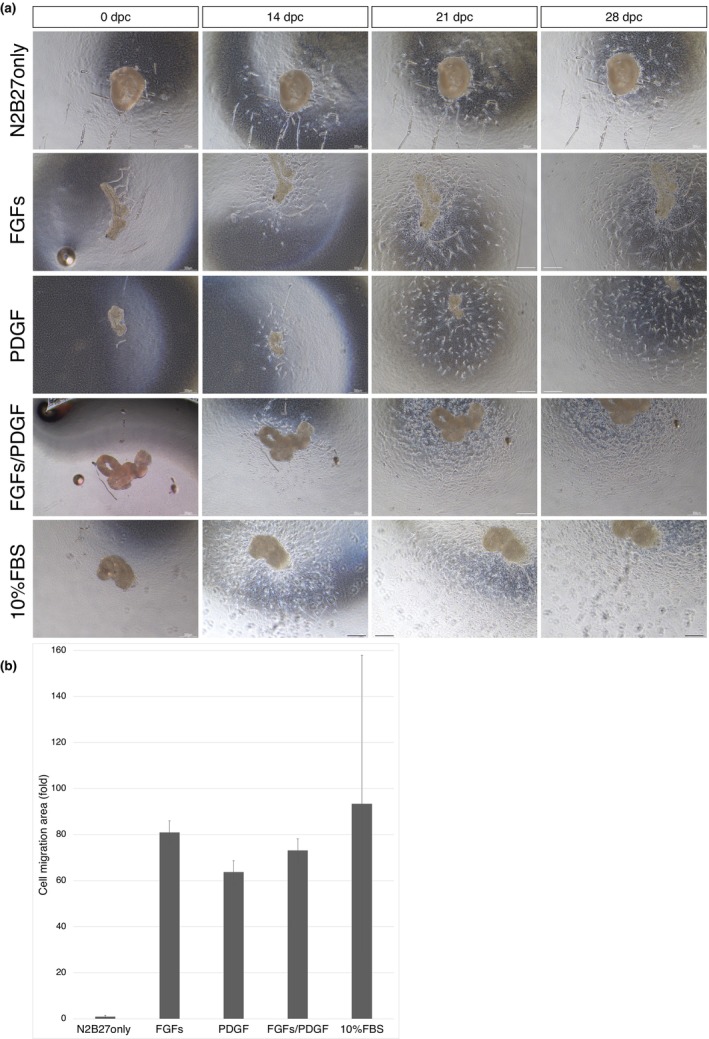
The range of cell migration was significantly expanded by adding regeneration‐related factors. (a) Cell migration over 0–28 days post‐ cultured (dpc). Bright field image. Scale bar, 300 μm. (b) Cell migration range. The influence of each factor was calculated using the migration area of the control as the reference value. *n* = 3. Bars: SE.

Cells started migrating on days 10–14 from the time of inoculation in the culture, and the extent of migration expanded rapidly from day 21 to day 31 (Figure 2a). At the start of migration, fibroblast‐like cells with long filopodia were abundant, but cells with elliptical nuclei filled the gaps in the middle. Tendon‐specific fibroblasts account for 95% of tendon cells (Li et al., 2021), and in wound healing, fibroblasts are thought to be activated by growth factors. The factors employed in this study were FGFs (FGF2 + FGF8) and PDGF, which may have activated fibroblast migration.

In contrast, no expansion of the migration range was observed in the cells cultured in basic medium without regeneration‐related factors. The tendons responded to regeneration‐related factors, indicating that the addition of these factors promoted cell migration (Figure 2b). The cell migration area in the FGFs‐, PDGF‐, and FGFs/PDGF‐supplemented media showed an 81‐fold, 63‐fold, and 73‐fold increase, respectively, compared to the negative control. Thus, the addition of regeneration‐related factors quantitatively promoted cell migration; however, comparison of the cell migration areas in each medium, including serum‐containing medium, showed no significant differences with those in media containing regeneration‐related factors.

### 3.3 Effects of regeneration‐related factors on cell proliferation

**FIGURE 3:**
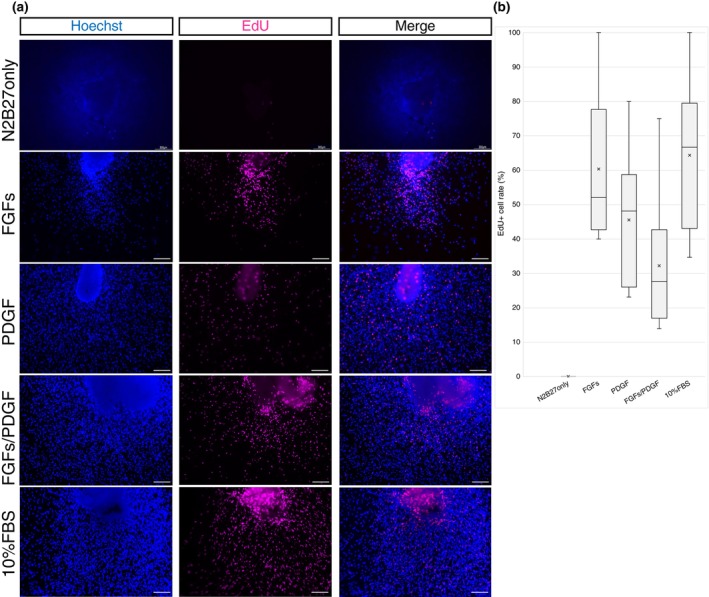
Active cell proliferation was confirmed by adding regeneration‐related factors. (a) EdU assay results for tendons cultured in media containing regeneration‐related factors. Cell proliferation was detected based on EdU incorporation (magenta) and Hoechst staining (blue) in the tendon. A reduced presence of Hoechst‐positive cells is observed, suggesting that cell viability might be reduced due to the absence of growth factors during prolonged culture of the explanted tissue in N2B27 medium only. Scale bar, 300 μm. (b) Percentage of EdU‐positive and Hoechst‐positive cells. Vertical lines indicate minimum and maximum values. Asterisks indicate mean values and horizontal bars indicate median values. *n* = 3.

The EdU assay was performed to investigate whether tendon‐derived cells had proliferative potential responding to regeneration‐related factors. To determine this, tissues were cultured in different media for 31 days, when cell migration was most pronounced. The collected tissues were cultured for 72 h in culture medium supplemented with EdU and fixed. EdU‐positive proliferating cells were not found in or outside the tissue in the basic medium (Figure 3a). In contrast, EdU‐positive cells were found in the tissue cultured in medium to which regeneration‐related factors were added. Therefore, cells that responded to the addition of regeneration‐related factors contained cells with proliferative potential. The percentage of EdU‐positive and Hoechst‐positive cells might be higher in tissues cultured in medium with only FGFs (Figure 3b). This indicated that the influence of each regeneration‐related factor changed not only the range of cell migration but also proliferation. As a trend, it can be observed that the proliferation‐promoting effect of FGF is strong. However, due to variability in the results, the difference in proliferative capacity among cells cultured with different regeneration‐related factors is not statistically significant.

### 3.4 Induction of chondrogenic differentiation by adding BMP7

**FIGURE 4:**
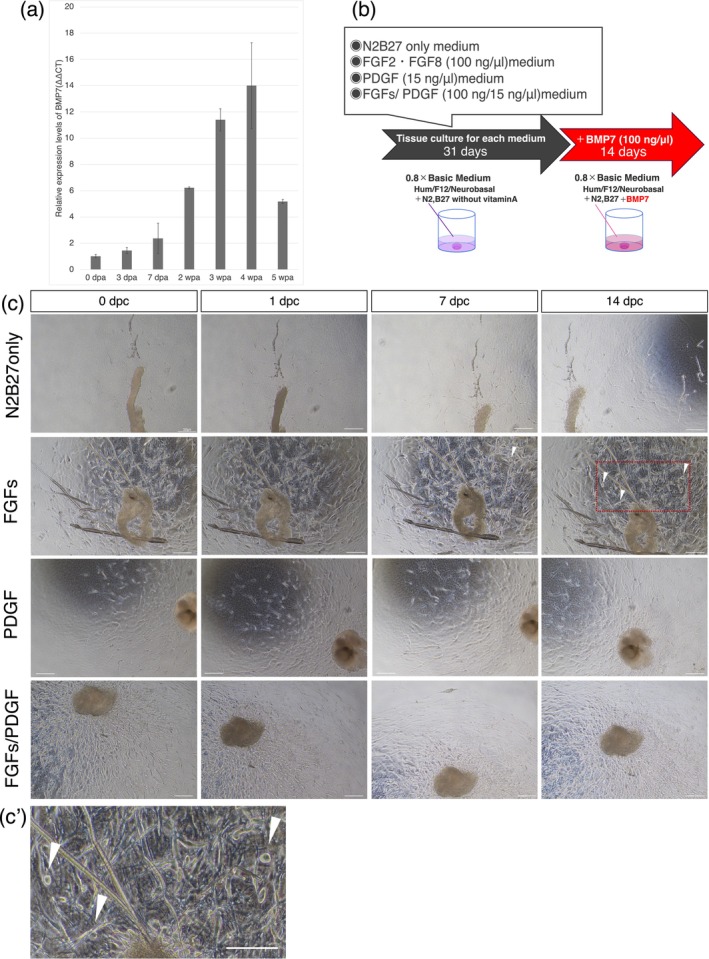
The effect of BMP7 on cells that proliferated and migrated in response to regeneration‐related factors. (a) Quantitative RT‐PCR results. This graph indicates the relative gene expression levels of BMP7 in the joint regeneration blastema samples (0 days post‐amputation to 5 weeks post‐amputation). Bars: SE. (b) The schematic of the tissue culture system. (c) Cell migration after BMP7 treatment. Bright field image. White arrowheads indicate chondrocyte‐like cells. Scale bar, 300 μm. (c′) A magnified view of the area surrounded by the red box in panel (c). Bright field image. White arrowheads indicate chondrocyte‐like cells. Scale bar, 300 μm.

BMP7 is involved in the maintenance and repair of cartilage homeostasis and plays an important role in the differentiation of mesenchymal cells into cartilage and hard bone. The expression of the BMP7‐encoding gene was analyzed by quantitative PCR during in vivo joint regeneration in *P. waltl*. Its expression level gradually increased, and at 4 weeks post‐amputation, it reached a peak (Figure 4a). Thus, BMP7 was added to culture media after 31 days of incubation, following removal of the FGFs‐ or PDGF‐containing medium (Figure 4b). After 14 days of incubation in BMP7‐containing medium, round‐shaped chondrocyte‐like cells appeared in the culture medium stimulating proliferation and migration with FGFs (white arrowheads in Figure 4c,c′). Such round‐shaped cells were not observed in other culture media.

**FIGURE 5:**
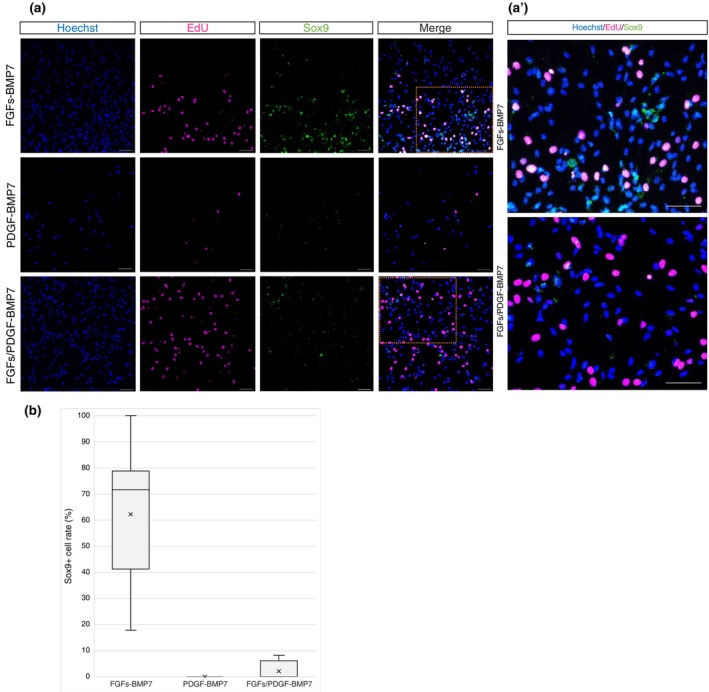
FGFs–BMP7‐supplemented medium induces differentiation into Sox9‐positive chondrocyte‐like cells. (a) Immunofluorescence cytochemistry of the tendon cultured with BMP7 culture medium. Cell proliferation was detected based on EdU incorporation (magenta); green indicates the cartilage marker Sox9 and blue indicates Hoechst staining. Scale bar, 100 μm. (a′) Magnified views of the areas surrounded by the orange boxes in panel (a). Scale bar, 100 μm. (b) Percentage of Sox9‐positive and Hoechst‐positive cells. *n* = 3.

To confirm chondrogenic differentiation, Sox9, a chondrocyte‐specific transcription factor, was detected by immunostaining (Figure 5a,a′). Although intact tendons and tendon‐derived cells that proliferated upon stimulation by FGFs or FGFs/PDGF could not express Sox9 before adding BMP7 (Figure S1), in the medium supplemented with FGFs and BMP7, Sox9‐positive cells emerged. This was particularly pronounced in the FGFs–BMP7‐supplemented medium, in which also morphological changes were observed, such as the development of larger, chondrocyte‐like round cells (Figure 4c′). Sox9 accumulates in the nucleus, and its signal is confined to the nucleus. Upon closer examination of the signal in Figure 5a′, as shown in Figure 5a, it becomes evident that there is some noise outside the nucleus. To precisely count the cells that have differentiated into chondrocyte‐like cells, we tallied the double‐positive cells stained with both Hoechst and anti‐Sox9 (Figure 5a′). Counting of Sox9‐positive cells revealed that, on average, 62% of the cells expressed Sox9 (Figure 5a′,b). Interestingly, some of these cells also tested positive for EdU, suggesting that Sox9‐positive cells may still maintain proliferative activity (Figure 5a, a').

Conversely, in the case of tendon‐derived cells that proliferated in the medium containing PDGF, Hoechst‐ and Sox9‐double‐positive cells were very rare, although nonspecific signals outside the nucleus were detected (Figure 5). Thus, it was demonstrated that the response to BMP7 differed based on the regeneration‐related factors added to the medium and their combination; the FGFs–BMP7‐supplemented medium induced differentiation into Sox9‐positive chondrocyte‐like cells. It was also suggested that differentiation of the chondrogenic lineage might be suppressed in the PDGF‐containing medium (Figure 5).

Quantitative RT‐PCR analysis using cells cultured in the medium supplemented with FGFs and FGFs/PDGF showed that the expression levels of cartilage‐related genes (*sox9* and *Col2a1* [Type IIa collagen]) were increased in cells cultured with only FGFs (Figure S2). Interestingly, the expression levels of tendon‐related genes such as *scx*, *tnmd* (tenomodulin), and *Col1a* (Type Ia collagen) were also increased in FGFs–BMP7‐supplemented medium. This suggests that the addition of BMP7 might be responsible for inducing chondrocyte differentiation in some cells that proliferated and migrated in response to FGFs. In contrast, the migrating cells in the PDGF‐containing medium might proliferate as fibroblastic cells (Figure 4c).

## 4 DISCUSSION

In this study, a *P. waltl* tendon tissue culture system was developed, which can be used to confirm their responsiveness to regeneration‐related factors. Tissue culture in *P. waltl* has been difficult in the past because of its large genome size (Brown et al., 2022) and the abundance of extracellular matrix (ECM) (Makanae et al., 2014). For example, in mice, migration was observed within 3 days even in 0.1% gelatin‐coated solution, but in newts, migration took more than a month. Fibronectin coating, which has already been reported as a joint cartilage induction medium (Lin et al., 2023), was also examined, but it did not accelerate tendon cell migration. In particular, the tendons took more than 2 months due to their low cell numbers. However, the use of a serum‐free laminin‐based cell culture substrate coating significantly improved migration initiation. This overcame the disadvantages of slow growth in a serum‐free medium and made it possible to more clearly demonstrate the responsiveness to each factor (Figures 1 and 2). In addition, this tissue culture system can be easily applied to observe reactivity and differentiation induction in other periarticular tissues under different factor combinations. It is speculated that this approach can contribute to the study of periarticular tissues.

During regeneration, FGFs and BMPs are secreted in the wound epithelium, which is known to induce blastema formation. In axolotl, FGF and BMP combinations are known to form blastemas instead of injury healing in damaged skin (Makanae et al., 2014; Makanae et al., 2016; Satoh et al., 2011; Satoh et al., 2022). BMP signaling input alone could not transform skin wound healing into limb formation, whereas FGF signaling input alone was reported to induce blastema but not limb formation (Makanae et al., 2014). In early limb bud development, BMPs are required for the induction of FGFs in the apical ectodermal ridge, but are later downregulated (Vincent et al., 2020). In this study, we focused on the addition period of FGFs and BMP7 to the medium to clearly understand their functions (Figures 2, 3, 4, 5). Differentiation into chondrocytes was observed only in the medium containing FGFs and BMP7 (Figures 4 and 5). This suggests that FGFs and BMP7 are likely to be joint regeneration‐related factors in *P. waltl*.

In contrast, an expanded range of cell migration and proliferation was observed in PDGF medium and FGFs/PDGF medium (Figures 2, 3, 4). In axolotl, PDGF‐BB is an activator of fibroblast migration essential for blastema growth or morphogenesis, and PDGF signaling is vital for connective tissue cells to migrate beyond the amputation plane in the first step to form the blastema (Currie et al., 2016). This suggests that cells responding to PDGF might interact with BMP7 but inhibit differentiation (Figure 5). Similar observations were made in frogs, mice, and chickens (unpublished data). However, if the differences in regenerative ability between these species are due to differences in the area of articular cartilage with and without secondary ossification, it is possible that they might not be able to fulfill their potential due to low availability of resources. Further analysis is required, but if this is the case in all animal species, it suggests that joint regeneration might be possible in non‐regenerating animals in the future.

Amputation of the limbs of urodele amphibians is known to cause de‐differentiation of the remaining parts (Satoh et al., 2011). The blastema cells produced by the de‐differentiated cells lose their localization once they are derived, but it is still debated whether they retain memory for their cell fate. It has been shown that in the dermis, they can undergo differentiation and contribute to the cells of the cartilage (Kragl et al., 2009; Satoh et al., 2022).

**FIGURE 6:**
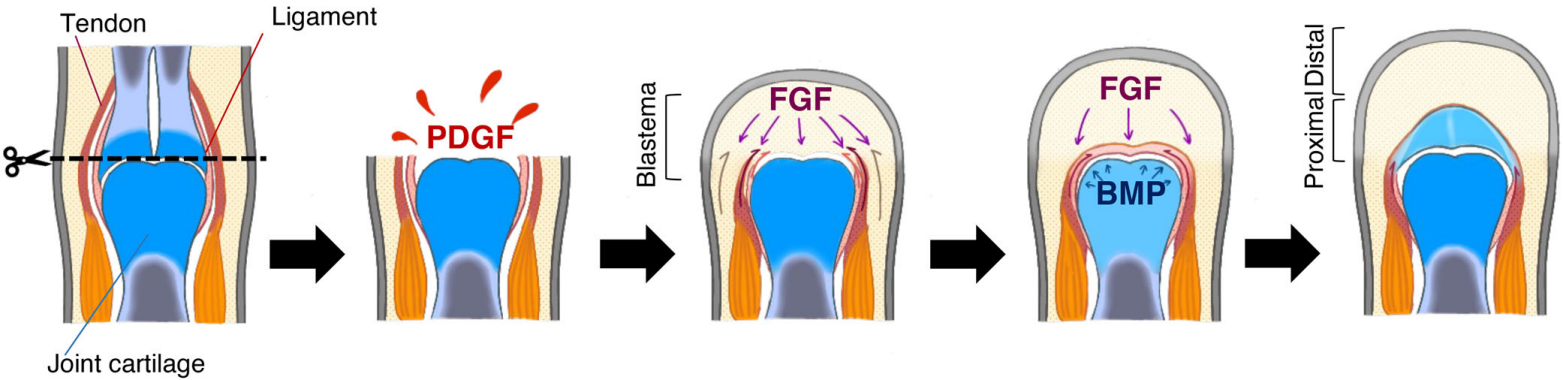
Models of joint cartilage regeneration involving tendons. Since amputation induces platelet‐derived growth factor (PDGF) expression, fibroblast growth factor (FGF) signaling induces tendon de‐differentiation and cell proliferation, and the addition of BMP induces differentiation into cartilage, tendon‐derived cells may contribute to joint cartilage in the blastema. The regenerating joint cartilage reconstructs its structure while surrounded by tendons/ligaments and is then connected to the regenerative bone structure on the distal side.

The origin of the chondrocytes present within the regenerating cartilage is not restricted to the chondrocytes of the remaining part but might also be derived from the periarticular tissues (Tsutsumi et al., 2015; Tsutsumi et al., 2016). The results of the EdU assay, with the addition of regeneration‐related factors, showed that cell proliferation was active in tendon tissue (Figure 3). With the addition of regeneration‐related factors, some of the tendon cell populations de‐differentiated in the same way as a blastema, and cell proliferation occurred within the tissue. Progenitor cells of both chondrocytes and tendon cells, originating from the lateral plate mesoderm, express both the cartilage marker gene *sox9* and the tendon marker gene *scx* during development; these cells differentiate into tendon cells and chondrocytes to form the junction between cartilage and tendon tissue (Sugimoto et al., 2013). In *X. laevis*, functional spike structures capable of flexion and extension can be regenerated by amputation at the elbow joint level (Tsutsumi et al., 2016). In the functional spike, it has been shown that tendon progenitor cells begin to differentiate in the mesenchyme of the regenerating area extension of the residual tendon, thereby connecting the residual muscle to the joint cartilage of the spike. Therefore, the Sox9‐positive chondrocyte‐like cells identified in the FGFs–BMP7 medium in this study were considered tendon‐derived cells that had returned to the multipotent state by regeneration‐related factors such as FGFs but were transdifferentiated into chondrocytes in response to BMP7. In the case of lens regeneration, pigmented epithelial cells (PECs), stimulated to proliferate by FGF, are known to differentiate into lens cells (Hyuga et al., 1993). The intermediate state cells that de‐differentiated are multipotent, and neither PEC‐ nor lens‐specific genes are expressed (Agata et al., 1993). It was also observed that basic FGF alone only promotes cell proliferation, whereas the combination of phenylthiourea and FGF promotes cell proliferation and de‐differentiation in ocular cells (Hyuga et al., 1993; Itoh & Eguchi, 1986). In the present study, we found that tendon‐derived cells that proliferated upon stimulation by FGFs can respond to BMP, differentiating into Sox9‐positive chondrocytes; FGF might promote not only cell proliferation but also cellular competency to be able to differentiate into chondrocytes, suggesting that the combination of FGF and BMP is important for the transdifferentiation of chondrocytes from tendon‐derived cells. Our recent study using *Xenopus* as a model system suggested that FGF can induce matrix metalloproteinase expression in remaining articular cartilage and that BMP might be secreted from the remaining articular cartilage via ECM degradation (Matsubara et al., 2023). Combining these results, it is postulated that functional joints are regenerated through a scheme similar to that summarized in Figure 6. With FGF stimulation, new joint cartilage is formed within the tissue extending from the tendon, released by BMP from the residual joint cartilage. This makes it possible to understand the robust connection between the residual muscles and the newly regenerated joint cartilage.

To validate these in vivo schemes, we are planning to conduct in vivo cell fate tracking using genetically modified Iberian ribbed newts (*P. waltl*). Recent advances in next‐generation sequencing technology have enabled the functional analysis of genes with the construction of gene catalogs and databases of *P. waltl* (Matsunami et al., 2019). Transgenesis and efficient knockout methods using CRISPR/Cas9 (Hayashi et al., 2013; Suzuki et al., 2018; Takeuchi et al., 2022) have also been developed. Currently, we are generating transgenic lines that fluorescently label *scx*‐positive cells permanently for cell fate tracing. Combining these findings with the tissue culture system used in this study may serve as a new tool to investigate the mechanism of joint regeneration in newts.

## AUTHOR CONTRIBUTIONS

KA conceived the study. NS performed the major experiments. NS and KA discussed the results and wrote the paper.

## Supporting information


**Figure S1.** Cells that migrated and proliferated upon stimulation by FGFs before adding BMP7 did not express Sox9. Green indicates Sox9 expression; Hoechst staining is shown in blue. Scale bar, 100 μm.
**Figure S2.** Quantitative RT‐PCR results. This graph indicates the relative expression levels of the tissue culture samples (FGFs‐BMP7, FGFs/PDGF‐BMP7). SE bars are not shown due to the limited RNA yield (*n* = 1 only). This scarcity stems from the minimal RNA extraction capacity from cultured tendon tissue cells.
