# Supplementary material for: FGF‐stimulated tendon cells embrace a chondrogenic fate with BMP7 in newt tissue culture
Source: Dev Growth Differ. 2024 Feb 11;66(3):182–93. doi: 10.1111/dgd.12913 (PMC11457504; doi:10.1111/dgd.12913)
Supplement: Supplementary file 1 — Figure S1. Cells that migrated and proliferated upon stimulation by FGFs before adding BMP7 did not express Sox9. Green indicates Sox9 expression; Hoechst staining is shown in blue. Scale bar, 100 μm. Figure S2. Quantitative RT‐PCR results. This graph indicates the relative expression levels of the tissue culture samples (FGFs‐BMP7, FGFs/PDGF‐BMP7). SE bars are not shown due to the limited RNA yield (n = 1 only). This scarcity stems from the minimal RNA extraction capacity from cultured tendon tissue cells. [file DGD-66-182-s001.pdf]

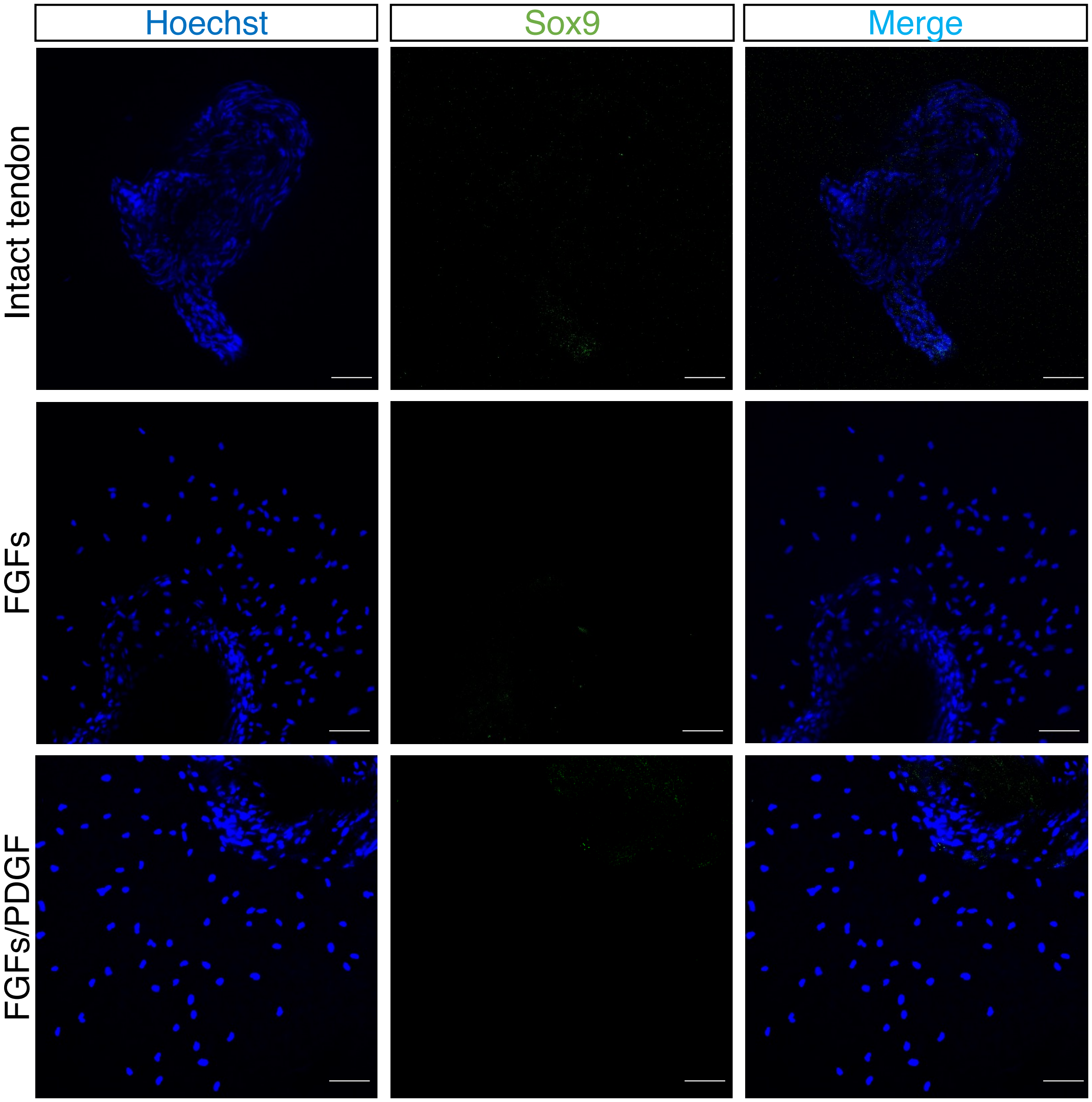

FIGURE S1. Cells that migrated and proliferated by FGFs before adding BMP7 did not express Sox9. Result of Immunofluorescence Cell Cytochemistry (IFC). Sox9 expression (Green) was stained with Hoechst (Blue) in the tendon with each tissue culture. Scale bar, 100 μm.

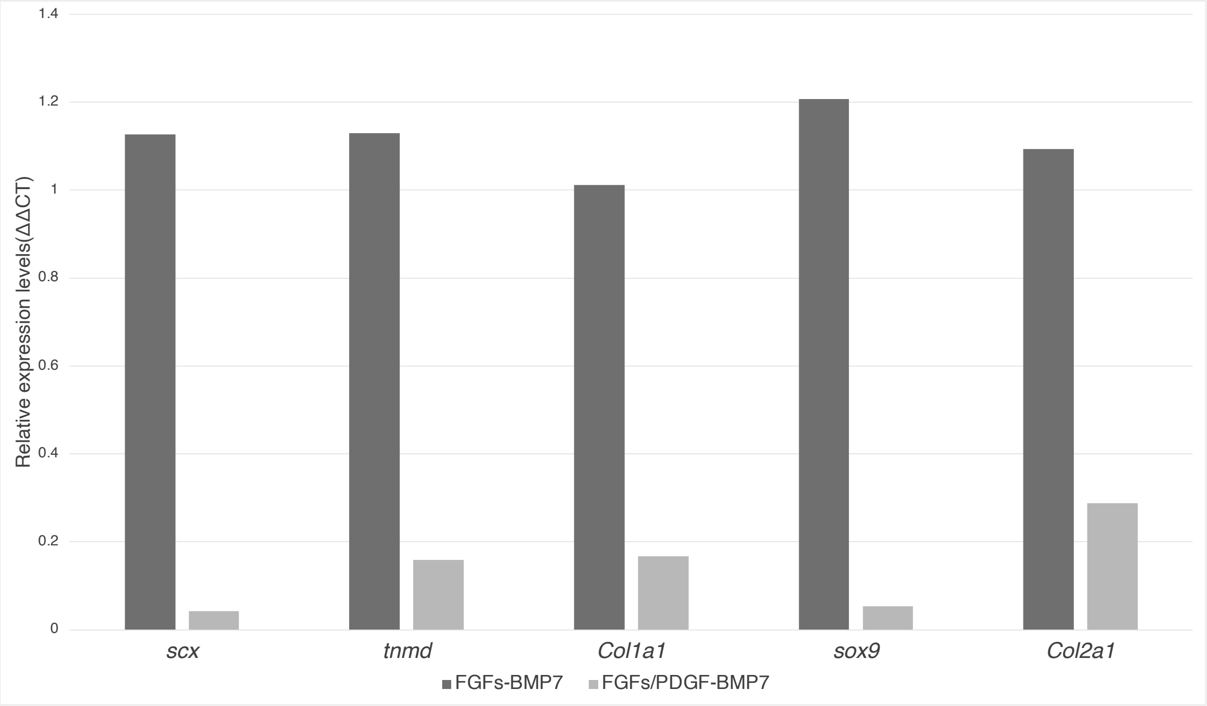

FIGURE S2. Result of quantitative RT-PCR. This graph indicates the relative expression patterns of the tissue culture samples (FGFs-BMP7, FGFs/PDGF-BMP7). The limited RNA yield, with only n=1 available, resulted in a small dataset, rendering the use of S.E. bars inappropriate for display. This scarcity stems from the minimal RNA extraction capacity from cells cultured of tendon tissue.
